# Data Innovation, Program Implementation, and Community Action (DIPICA) Observatory for Surgical, Anesthesia, and Obstetric (SAO) Care in India

**DOI:** 10.1186/s12919-025-00351-3

**Published:** 2025-11-03

**Authors:** Shirish Rao, Uma Gupta, Siddhesh Zadey, Lovenish Bains, Dhruva Ghosh, Joao Ricardo Nickenig Vissoci, DIPICA Collaborative, Shirish Rao, Uma Gupta, Siddhesh Zadey, Lovenish Bains, Dhruva Ghosh, Joao Ricardo Nickenig Vissoci, Aaradhana Vaghela, Aiman Perween Afsar, Anchal Dhiman, Anoushka Arora, Anurag Mishra, Chaitanya Reddy, Chinmayee Swain, Gnanaraj Jesudian, Harsh Thakkar, Janet Martin, Kalpana Pawalia, Kapil Dev Soni, Keyur Buch, Lalit Gupta, Madhurima Vuddemarry, Maithili Kukade, Naveen Sharma, Nishikant Singh, Ojaswi Phal Desai, Padmavathy Krishna Kumar, Pratheeba John, Preeti Kumar, Priyansh Nathani, Rahul M. Jindal, Rajesh Mehta, Rajna Mishra, Rakesh Garg, Ritika Shetty, Rituparna Sengupta, Rodney Preetham Vaz, Samruddha Kulkarni, Sanjay Nagral, Shikha Sharma, Shreyas Patil, Sudheer Kumar Shukla, Suraj Bhor, Tej Prakash Sinha, Tushar S. Mishra

**Affiliations:** 1 Association for Socially Applicable Research (ASAR), Pune, Maharashtra India; 2 Department of Surgery, BDBA Municipal General Hospital, Mumbai, Maharashtra India; 3 Department of Epidemiology, Mailman School of Public Health, Columbia University, New York, NY USA; 4 GEMINI Research Center, Duke University School of Medicine, Durham, NC USA; 5 Dr. D. Y. Patil Dental College and Hospital, Dr. D. Y. Patil Vidyapeeth, Pune, Maharashtra India; 6 Department of General Surgery, Maulana Azad Medical College, New Delhi, India; 7 India Hub, NIHR Global Health Research Unit On Global Surgery, Christian Medical College, Ludhiana, Punjab India; 8 Duke Global Health Institute, Durham, NC USA; 9 Department of Surgery, Duke University School of Medicine, Durham, NC USA; 10 Department of Emergency Medicine, Duke University School of Medicine, Durham, NC USA; 11 Department of Neurosurgery, Duke University School of Medicine, Durham, NC USA

**Keywords:** Global Surgery, Health planning and policy, Financing, Workforce, Advocacy, India, LMIC

## Abstract

The Data Innovation, Program Implementation, and Community Action (DIPICA) Observatory for Surgical, Anesthesia, and Obstetric (SAO) Care in India convened in December 2024 in New Delhi. This two-day program brought together experts from global surgery, public health, health policy, and clinical practice to address planned challenges in SAO care, focusing on data-driven approaches, sustainable financing, and workforce development. The discussions emphasized leveraging data to strengthen SAO care systems by creating practical data products for localized planning and community engagement. Participants advocated for dynamic observatories to support data-driven research and policy implementation, making SAO care more accessible and efficient. Sustainable financing emerged as a key focus, with experts discussing innovative approaches that move beyond conventional models. Community-based solutions, decentralized funding, and performance-based models were discussed as strategies to optimize resource allocation. Establishing partnerships involving government, private, and community sectors was highlighted as crucial for achieving financial sustainability, particularly in underserved areas. Workforce development discussions addressed competency-based training and task-sharing initiatives to reduce disparities. The attendees agreed on the need for structured training to enhance surgical skills, especially for general practitioners in rural areas. Regulatory policies on task-sharing and task-shifting were considered essential to maintaining care quality while expanding access. Improving trauma care and emergency response through targeted training and rural workforce retention also remained key priorities. Advocacy efforts focused on integrating SAO care into national health priorities through data-driven communication and public engagement. The observatory aims to sustain progress through collaborative research, advocacy, and policy dialogues to ensure equitable, accessible, and high-quality SAO care across India.

**Supplementary Information:**

The online version contains supplementary material available at 10.1186/s12919-025-00351-3.

## Introduction

India has made significant progress in health in the past 77 years, as witnessed by substantial reductions in infant and maternal mortality, gains in life expectancy, and improvements in access to care [1]. Despite uneven distribution, there is a well-documented growth of a well-trained health workforce, remarkable uptake of public health interventions, an increase in the number of tertiary hospitals, and implementation of the National Health Mission and Ayushman Bharat-Pradhan Mantri Jan Arogya Yojana (PMJAY) [1]. However, there are areas which need major focus such as shortage of trained SAO health workers, lack of standardization and protocols for quality healthcare, disparities in access across urban and rural areas, and high out-of-pocket expenditures [2]. Further success will require a national policy to collect outcome data for SAO care problems and culturally appropriate solutions.

The Lancet Commission on Global Surgery (LCoGS) noted the significant gaps in access to surgical, anaesthesia, and obstetric (SAO) care as a neglected problem in global health [3]. The ‘Karad Consensus Statement’ (2016), led by the Association of Rural Surgeons of India, further underscored India-specific challenges [4, 5]. Significant strides have been made in recent years, including mapping SAO indicators for India, innovating frugal equipment for minimally invasive essential surgeries, and upskilling healthcare workers to engage in various aspects of SAO care delivery [6, 7]. Ensuring timely access to safe and affordable SAO care for all Indians would have positive societal spillover, including poverty reduction [8, 9]. However, addressing challenges in SAO care requires data-driven insights with real-world program implementation to create practical and scalable solutions. Integrating SAO care with broader public health priorities like primary health care (PHC), universal health coverage (UHC), maternal and child health (MCH), and the National Health Mission in the Indian context can make it more effective and sustainable.

The Data Innovation, Program Implementation, and Community Action (DIPICA) for SAO Care 2024 Meeting aimed to: a) Discuss the use of data-driven approaches to monitor and evaluate SAO care program implementation to improve population health. b) Understand the facilitators and barriers in using data to inform policies and strategies that can help universalize SAO care and strengthen the Indian health system. c) Bring together interdisciplinary interest-holders from all sectors that can form a community for action to deliberate on and propose future research, advocacy, and implementation.

Tables 1 and 2 provide a brief overview of the meeting and the program agenda. In this manuscript, we describe the main themes discussed during the meeting, during which attendees formed a consensus on key challenges and strategic approaches related to SAO care.

**Table 1:** Meeting overview

**Fig. 1:**
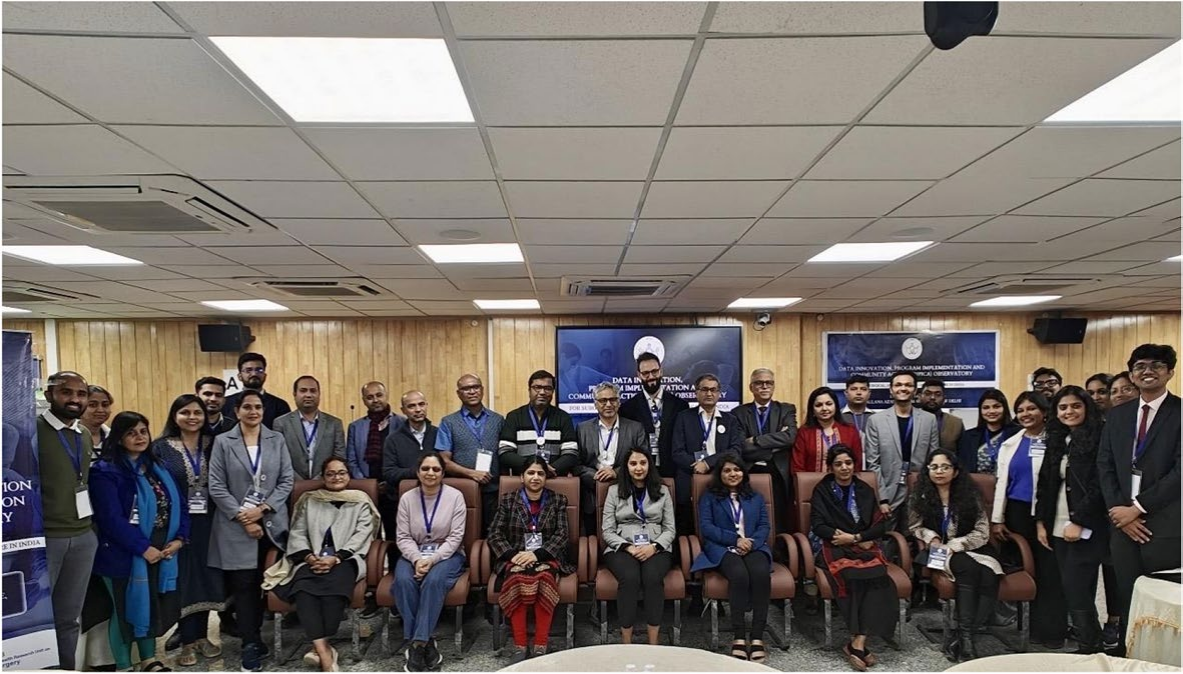
Group picture of DIPICA attendees—day 1

**Fig. 2:**
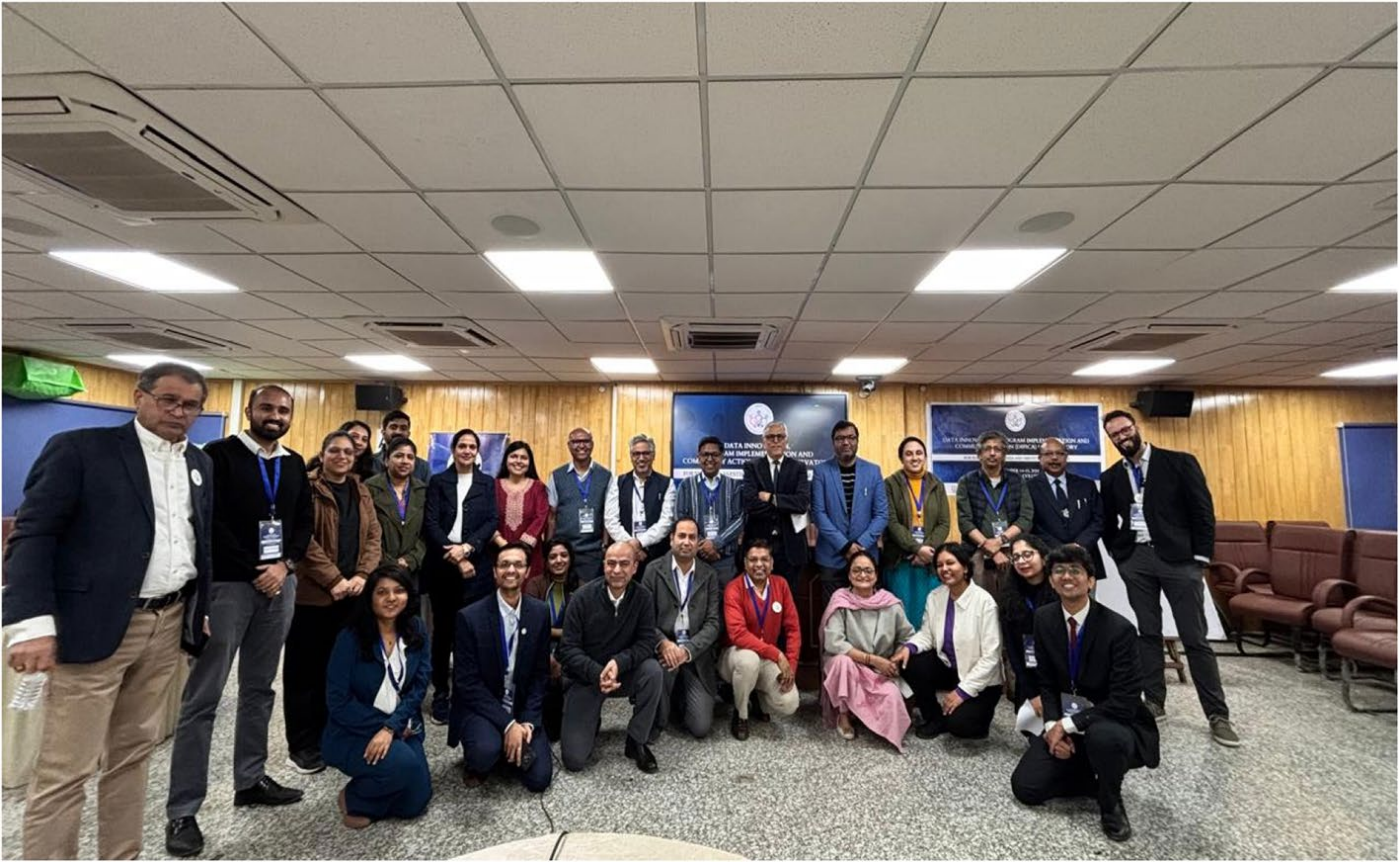
Group picture of DIPICA attendees—day 2

The ‘Data Innovation, Program Implementation, and Community Action (DIPICA) for Surgical, Anesthesia, and Obstetric (SAO) Care 2024’ meeting on December 14–15 was co-hosted by the Departments of Surgery, Maulana Azad Medical College (MAMC), New Delhi, Association for Socially Applicable Research (ASAR), NIHR Global Surgery Hub—India, and Duke Global Emergency Medicine Innovation and Implementation (GEMINI) Research Center at MAMC, New Delhi, and sponsored by Duke Global Health Institute (DGHI). It gathered over 50 experts in global surgery, public health, health policy, economics, advocacy, nursing, and medicine from leading institutions and think tanks including the National Health System Resource Centre (NHSRC), National Health Authority (NHA), Public Health Foundation of India (PHFI), Association of Rural Surgeons of India (ARSI), Health Systems Transformation Platform (HSTP), Centre for Health Equity Law and Policy (C-HELP) and academic institutions including MAMC, AIIMS New Delhi, AIIMS Jodhpur, AIIMS Bhubaneswar, and CMC Ludhiana. Experts from multiple WHO Regional Offices were also present in their capacity. Figures 1 and 2 show a group picture of all the meeting attendees on day 1 and day 2, respectively. This meeting is one of the first of its kind, bringing together a diverse group of experts to enhance SAO care from the health system and policy perspective in India
The meeting, organized by Dr Lovenish Bains, Dr Dhruva Ghosh, Mr Siddhesh Zadey, Dr Joao Ricardo Nickenig Vissoci, Dr Uma Gupta, and Dr Shirish Rao, focused on leveraging innovative data-driven approaches to enhance the implementation of the SAO care programs and improve population health. Discussions focused on the role of data, financing, workforce, advocacy, and policy for strengthening SAO care systems in India. Through consultation sessions held on both days, participants offered actionable insights on challenges and opportunities for data-driven SAO care delivery. Attending delegates shared their perspectives on SAO workforce development, unmet needs, robust data systems, and strategies to integrate SAO care into broader health policies for trauma, non-communicable diseases (NCDs), cancer, and maternal and child health programs for better implementation

**Table 2:** Agenda for DIPICA for SAO Care India meeting 2024

Day 1		
Time (IST)	Agenda	Speakers/Discussants
2:30—2:55 pm	Orientation • Opening remarks by hosts • Introducing the meeting agenda • Introducing the agenda for Day 1	Dr Dhruv Ghosh, Co-Director, India Hub, NIHR Global Health Research Unit On Global Surgery, Christian Medical College, Ludhiana Dr Lovenish Bains, Professor, Department of General Surgery, Maulana Azad Medical College, New Delhi Mr Siddhesh Zadey, Co-founding Director, Association for Socially Applicable Research (ASAR), Pune
3:00—4:20 pm	Data Innovations for SAO Care • Global SAO Care Movement • SAO Care Indicators for India • Surgical Preparedness of Hospitals • Using Data for Better Health • Open Discussion	Dr Dhruv Ghosh, Co-Director, India Hub, NIHR Global Health Research Unit On Global Surgery, Christian Medical College, Ludhiana Mr Siddhesh Zadey, Co-founding Director, Association for Socially Applicable Research (ASAR), Pune Dr Joao Vissoci, Director, Duke Global Emergency Medicine Innovation and Implementation (GEMINI) Research Center, USA
4:30—4:55 pm	High Tea & Networking	
5:00—5:55 pm	Pathways to Financing SAO Care in India • Financial Risks and Protections for SAO Care Panel Discussion: ◦ Different models of financing and evidence supporting these models ◦ Role of good data systems for monitoring and evaluation • Moderator Remarks • Open Discussion	Moderator: Dr Uma Gupta, Research Volunteer, Association for Socially Applicable Research (ASAR) Dr Gnanraj Jesudian, Past President, Association of Rural Surgeons of India (ARSI) Dr Pratheeba John, Associate Director—Health Financing & Planning, Health Systems Transformation Platform (HSTP), New Delhi Dr Keyur Buch, Independent Orthopedic Surgeon, Ahmedabad
6:00—6:55 pm	Consultation I: Financing and Policy for SAO Care in India • Orientation • Questions • Conclusion	Moderator: Dr Shirish Rao, Research Volunteer, Association for Socially Applicable Research (ASAR), Pune
Day 2		
9:30—10:15 am	Orientation • Recap of Day 1 ◦ Insights from Consultation I • Introducing the agenda for Day 2	Mr Siddhesh Zadey, Co-founding Director, Association for Socially Applicable Research (ASAR), Pune Dr Rajesh Mehta, Former Regional Advisor for child and adolescent health, WHO-SEARO, New Delhi
10:20—11:15 am	Data Initiatives for Improving Program Implementation for SAO Workforce Strengthening • SAO Workforce in India in Numbers • Panel Discussion on ◦ Indicators and targets for production and scale-up ◦ Task sharing/Shifting, Rural Surgery training • Moderator Remarks • Open Discussion	Moderator: Dr Shirish Rao, Research Volunteer, Association for Socially Applicable Research (ASAR), Pune Dr Gnanraj Jesudian, Past President, Association of Rural Surgeons of India (ARSI) Dr Keyur Buch, Independent Orthopedic Surgeon, Ahmedabad Dr Rakesh Garg, Professor, Department of Anesthesiology, Pain & Palliative Care, AIIMS New Delhi Dr Naveen Sharma, Professor and Head, Department of General Surgery, AIIMS Jodhpur Dr Tushar Mishra, Professor and Head, Department of General Surgery, AIIMS Bhubhaneshwar Dr Tej Prakash Sinha, Co-Director, WHOCC for Emergency and Trauma Care, AIIMS New Delhi Mrs Roopa Rawat, Regional Nursing Lead, WHOCC for Emergency and Trauma Care, AIIMS New Delhi
11:20—11:50 am	High Tea & Group Photograph	
12:00—12:25 pm	Using Data for Advocacy for SAO Care: What, How, and Who?	Moderator: Ms Shefali Malhotra, Research Consultant, Centre for Health Equity, Law & Policy (C-HELP), New Delhi Dr Sanjay Nagral, Director, Department of Surgical Gastroenterology, Jaslok Hospital, Mumbai Dr William Bhatti, Medical Superintendent, Christian Medical College, Ludhiana Dr Dhruva Ghosh, Co-Director, India Hub, NIHR Global Health Research Unit On Global Surgery, Christian Medical College, Ludhiana
12:30—12:55	Defining the unmet needs of surgical care in low-income and middle-income countries	Dr Lovenish Bains, Professor, Department of General Surgery, Maulana Azad Medical College, New Delhi Dr Anurag Mishra, Professor, Department of General Surgery, Maulana Azad Medical College, New Delhi Dr Deepa KV, Consultant, Department of Surgery, Max Hospital, New Delhi Dr Lalit Gupta, Professor, Department of Anesthesiology, Maulana Azad Medical College, New Delhi
1:00 pm—1:50 pm	Lunch	
2:00—3:20 pm	Facilitators and Barriers to Data-driven SAO Care Prioritization- Debate & Discussion	Moderator: Mr Siddhesh Zadey, Co-founding Director, Association for Socially Applicable Research (ASAR), Pune Dr Preeti Kumar, Vice President—Health Systems, Public Health Foundation of India (PHFI), New Delhi Dr Chinmayee Swain, Sr Consultant, National Health Systems Resource Centre (NHSRC), New Delhi Dr Ratan Shekhawat, Consultant, National Health Systems Resource Centre (NHSRC), New Delhi Dr Shikha Sharma, Consultant, National Health Systems Resource Centre (NHSRC), New Delhi Dr Kalpana Palwalia, Consultant, National Health Systems Resource Centre (NHSRC), New Delhi Dr Anchal Dhiman, Consultant, National Health Systems Resource Centre (NHSRC), New Delhi Dr Sonali Vaid, Technical Officer—Safe and Affordable Surgery, WHO-WPRO, Philippines
3:30—4:40 pm	Consultation II: Data Innovation and Workforce for SAO Care in India • Orientation • Questions • Conclusion	Moderator: Dr Uma Gupta, Research Volunteer, Association for Socially Applicable Research (ASAR), Pune
4:45—6:00 pm	Closing remarks • Future steps • Note of thanks • High Tea & Departure	Dr Lovenish Bains, Professor, Department of General Surgery, Maulana Azad Medical College, New Delhi Dr Joao Vissoci, Director, Duke Global Emergency Medicine Innovation and Implementation (GEMINI) Research Center, USA

### Role of data for SAO care

#### Data and data products on SAO care indicators in India

Mr Siddhesh Zadey addressed the nuances of translating data into tangible impact using the Lancet Commission on Global Surgery Indicators (LCoGS) [3, 10]. He underscored the need to map health indicators at a regional level and adjust goals to fit the actual health needs of each country to ensure sustainable and fair SAO care planning. Mr Zadey discussed the various data sources and methodologies ASAR used to gather accurate information at the national, state, and local levels and shared two key examples for India.

First, leveraging data from a 2021 National Institution for Transforming India (NITI) Ayog report, they mapped travel times to SAO care at district hospitals and analyzed changes in access when considering factors like the number of surgeons and surgical efficiency [11]. Second, using data from HMIS and NFHS, they analyzed trends in surgical volumes, focusing on major surgeries, to determine whether major or minor procedures drove global and national increases in surgical numbers—or were merely due to changes in reporting practices [12].

He also proposed two solutions to create data products useful to decision-makers at all levels: a dashboard that includes LCoGS and other SAO care indicators such as cesarean section rates, surgical sterilizations, blood banking performance, SAO spot density in medical college hospitals, etc., modelled at the district level, and a concise summary document, i.e., fact sheet, designed to present specific data points to policymakers and other interest-holders.

#### Development of a surgical preparedness index

Dr Dhruva Ghosh emphasized the role of surgery in improving health outcomes, where early and appropriate surgical interventions can significantly impact health demographics. He highlighted the need to integrate surgery into community health for universal access, rather than being confined to hospitals. A key focus of his work is the Surgical Preparedness Index (SPI), a tool developed during the COVID-19 pandemic to assess hospitals’ ability to sustain elective surgery during pandemics, natural disasters, or conflicts/wars [13]. The SPI was developed in the context of pandemic preparedness through international collaboration, including hubs in Africa, Mexico, the Philippines, and India. The SPI comprises 23 indicators related to facility reliability, staffing, prioritization, and resource availability. It aims to assess whether hospitals are prepared to continue essential elective surgeries, such as cancer surgery, during periods of stress, such as during pandemics, natural disasters, or warfare. Results showed that hospitals with higher SPI scores were able to maintain better elective surgery volume ratios during the pandemic [13]. Dr Ghosh highlighted the importance of surgical preparedness and timely interventions, especially in resource-constrained settings. He explained that the SPI captures the unique challenges and resource constraints by facilitating data comparison on preparedness across diverse healthcare contexts in countries across different income groups. Dr Ghosh acknowledged a key limitation of the SPI—its current focus on system preparedness rather than the quality and safety of surgeries.

#### Usage of secondary data for better health

Dr Joao Ricardo Nickenig Vissoci discussed the potential of using secondary data to improve health outcomes and access to care through a community-based approach. He proposed the concept of observatories—dynamic, long-lasting data hubs that evolve with community engagement to support data-driven research, community action, and policy implementation aimed at enhancing healthcare access, financing, and quality. Dr Vissoci highlighted that DIPICA was designed as an observatory to leverage data-driven strategies for monitoring and implementing SAO care in India. He gave examples from across the world where such initiatives have been integral in advocacy for critical community needs, including improved diagnostic accuracy and healthcare infrastructure. At Amazon, a satellite-based network system for viral biosurveillance, identification, and laboratory classification known as VIVERA was developed [14]. This was followed by SAVING, a decentralized AI model for antivenom distribution based on the location of healthcare facilities to ensure access to antivenom within six hours [15]. He mentioned another innovative solution from Brazil connecting trauma patients, taxi drivers, and hospitals [16]. Additionally, he discussed a virtual reality-based task-sharing model for neurosurgery in Tanzania, enabling remote guidance from specialized surgeons to non-specialists [17].

### Financing SAO care

Dr Uma Gupta discussed ASAR’s current work on assessing the role of Ayushman Bharat’s Pradhan Mantri Jan Arogya Yojana (PM-JAY) in enhancing SAO care coverage. She highlighted findings from the National Sample Survey 2017–2018, which revealed that 86% of households incurring surgical costs face catastrophic health expenditures, and 73% suffer impoverishment [18]. She emphasized the heterogeneity in financial risks and access to SAO care, which varies across states, districts, and vulnerable populations. Dr Gupta further elaborated on PMJAY, noting that while it has significantly enhanced surgical coverage, it faces challenges such as inequities in gender and regional access [18, 19]. For instance, pediatric surgery and polytrauma are underrepresented in pre-authorization approvals compared to obstetrics and surgical oncology.

The panel discussion on Financial Risks and Protections for SAO Care focused on strategies and models to make SAO care cost-effective. Dr Nilesh Chandra discussed the recent reorganization at the Indian Council of Medical Research (ICMR) to focus on delivering research for health schemes. He encouraged interest-holders to leverage ICMR funding opportunities for innovative projects in SAO care. Dr Chandra stressed the importance of identifying gaps and supporting projects to improve resource allocation and service delivery.

Dr Gnanraj Jesudian shared perspectives on financing rural and remote SAO care. He elaborated on his work with the Kukna tribes in Gujarat, where a lack of doctors in the 1980 s inspired efforts to develop innovative models for rural surgery [20]. These included surgical camps, low-cost innovative technologies such as gasless laparoscopy devices and portable cystoscopy equipment, and regional training programs [21]. The low-cost innovations not only reduced costs but also required simpler training, making them ideal for resource-constrained settings. They have also been adopted at Kabale University in Uganda [22, 23]. While models like the Burrows Memorial Christian Hospital (BMCH) initiative in Assam have successfully increased surgical access, he elaborated on challenges such as delayed payments under PM-JAY and workforce shortages that hinder sustainability [24].

Dr Pratheeba John discussed the importance of cost-effectiveness in financing SAO care, particularly in India’s resource-constrained settings. She recommended taking inspiration from models from other low- and middle-income countries (LMICs), such as Thailand’s universal access program, and tailoring them to India’s diverse needs [25]. She also advocated for transitioning from input-driven to performance-based financing. She explained how decentralized funding models empower facilities to address local needs effectively while regional specialization ensures efficient resource allocation. Dr John called for partnerships involving the government, the private sector, and community organizations to pool resources and establish short, medium, and long-term plans to optimize resources and address structural limitations.

### SAO care workforce

Dr Shirish Rao opened the panel discussion on the SAO care workforce in India, highlighting key challenges related to workforce distribution, training, and competency. India faces a significant shortage of SAO care providers, with a workforce density of 6.5 per 100,000 population in 2009, which is far below the global benchmark set by the LCoGS [26, 27]. Despite efforts to increase postgraduate and super-speciality training seats, production capacity remains insufficient, with a skewed distribution favouring southern and western states while central and northeastern regions remain underserved [28]. Further, Dr Lovenish Bains, Dr Anurag Mishra, and Dr Deepa KV discussed findings from their ongoing analysis of unmet needs of SAO care in LMICs in the context of LCoGS indicators, highlighting that about 80% of unmet needs of the workforce could be solved by bridging the workforce gap.

#### Competency-based training of workforce

Panellists, including Dr Rakesh Garg, Dr Sanjay Nagral, and Dr Gnanraj Jesudian, discussed alternative approaches to address workforce shortages, including the involvement of MBBS and AYUSH doctors using task-sharing and task-shifting models. They emphasized that merely increasing workforce numbers without adequate training and competency checks would not resolve underlying issues. Task sharing emerged as a viable solution throughout the discussion, elaborating on the potential and challenges of recruiting MBBS doctors in providing anaesthesia care and task sharing with non-physicians, including nurse anaesthetists [29]. The attendees agreed that clear distinctions between task sharing within the same cadre and task shifting to less-trained personnel should be based on competency skills rather than procedures.

Dr Naveen Sharma, Dr Tushar Mishra, and Dr Keyur Buch deliberated on defining competency benchmarks using data indicators to measure workforce effectiveness. They emphasized the need for structured competency frameworks that align with international best practices while considering India's unique healthcare landscape. They discussed initiatives for training and skill development in low-resource settings, including structured mentoring programs, standardized logbooks, and competency-based accreditation. The panellists stressed the importance of integrating continuous learning and conducting periodic evaluations to maintain workforce quality. They also highlighted the need for clear guidelines, robust regulatory oversight, and competency-based training to ensure patient safety and quality of care. The panellist proposed that regulatory bodies such as the National Medical Commission, National Board of Examinations in Medical Sciences, and AIIMS could provide unique accreditation to individuals who may not meet traditional educational standards but are capable of performing SAO care in a defined scope of practice with supervision and special licensing.

Dr Tej Prakash Sinha led the discussion on trauma care, focusing on workforce training to address the high injury burden in India [30]. Panellists discussed the need for targeted capacity-building efforts, particularly in rural and semi-urban areas where trauma care services are often inadequate. Dr Preethi Kumar and Dr Sanjay Nagral deliberated on point-of-care training for paramedical staff at primary care centres and an efficient referral network to address the burden of trauma due to road and railway traffic accidents. Dr Tushar Mishra emphasized the need to include hands-on trauma care training and simulation-based training for medical graduates.

Dr Tushra Mishra discussed the challenges faced by surgical training faculty, particularly in balancing mentoring with clinical duties. He noted that the extra hours required often prevent faculty members from dedicating enough time to teaching. To address this, he proposed a structured training model that balances and prioritizes mentorship and skill development alongside patient care. Dr Anurag Mishra added that working in rural areas also requires administrative skills, and the lack of these skills can deter surgeons from practising in such settings.

#### Workforce strengthening initiatives by the government of India

Dr Chinamyee Swain discussed the Aspirational District Program launched by the Government of India (GOI), which targets vulnerable areas with high healthcare needs [31]. This program involves five funded blocks aimed at improving healthcare delivery through mapping local healthcare facilities and offering financial incentives for doctors to improve retention. Dr Swain also spoke about the trauma care facilities developed at the primary care centres by the Ministry of Health and Family Welfare (MoHFW) in the hilly regions of Ladakh, Union Territory of Kashmir, which can act as a model for exceptions in the other areas of India.

Dr Kalpana Pawalia discussed the task-sharing and shifting initiatives under the MoHFW, like Comprehensive Emergency Obstetric and Newborn Care (CEmONC) and Life-Saving Anesthetic Skills (LSAS) training [32]. These allow non-specialist medical officers to perform essential maternal and newborn healthcare interventions, including cesarean sections and anesthesia administration in areas facing a shortage of specialist doctors. This approach improves access to emergency obstetric care and helps reduce maternal and neonatal mortality, aligning with India’s public health goals. She elaborated on the need to strengthen and support these existing programs, especially those designed for rural and far-reaching areas of the country.

They explained that the MoHFW provides technical and financial support to the States/UTs to strengthen the public healthcare system. This includes recruiting healthcare professionals in rural and underserved areas and upgrading medical equipment under the National Health Mission. Various financial incentives are provided, including a) ‘Hard area allowance’ to specialist doctors for residential quarters in rural areas; b) Honorarium to gynaecologists, paediatricians and anaesthetists as well as CEmONC and LSAS trained doctors for conducting cesarean sections in rural & remote areas; c) Salary negotiations for specialists in certain states including flexibility strategies such as “You Quote We Pay.” The National Health Mission also supports the multi-skilling of doctors to overcome the shortage of specialists.

#### Workforce regulations

Dr Preethi Kumar highlighted that national programs and policies often neglect surgical care, resulting in a healthcare workforce that lacks the necessary competency to provide surgical services [33]. Mrs Roopa Rawat stressed that self-regulation among professional bodies often impacts the implementation of national guidelines, particularly at the subnational level. She noted that the autonomy of various specialist medical societies leads to a lack of uniformity in training standards, creating barriers to improving care, particularly in rural settings. She also pointed out that inconsistent governance structures can lead to disparities and eventually compromise the quality of care.

A major theme of the discussion was the shift from task-sharing to collaborative care. Mrs Rawat emphasized the integral role of nurses in surgical teams, advocating for a model where healthcare professionals work together to provide holistic care rather than limiting nurses to task-specific roles. This approach would enhance teamwork and ensure that patients receive comprehensive care. Dr Vissoci concluded by stressing the importance of optimizing surgical capacity through decentralization and sharing of resources, which could reduce the burden on urban hospitals and improve access to care in rural areas.

### SAO care advocacy

Ms Shefali Malhotra moderated the panel discussion on SAO care advocacy, emphasising the need for distinct strategies to engage policymakers and the general public. Dr Sanjay Nagral and Dr Dhruva Ghosh mentioned that the healthcare system remains highly fragmented, with advocacy efforts often lacking cohesion and a unified voice. The resistance from professional associations to innovative models such as task-sharing further hampers progress, creating significant roadblocks to addressing workforce shortages [34]. Additionally, the lack of data accessibility for both policymakers and the public was highlighted as a major concern, with panellists emphasizing the need for simplified, easily understandable reports that effectively communicate the realities of SAO care gaps.

Dr William Bhatti elaborated on the importance of data-driven advocacy. Drawing from his experience with the Punjab State Government, he emphasized that policymakers are more receptive to well-structured advocacy messages backed by ethically sourced data, including service utilization, and the financial impact of unmet surgical needs. Advocacy efforts targeting policymakers must be tailored to align with national health priorities and political agendas. Dr Ghosh added on the need to complement quantitative data with qualitative narratives that capture the lived experiences of patients and healthcare providers. He stressed that effective advocacy requires sustained engagement with policymakers through policy dialogues, participation in government-led consultations, and collaboration with interest holders like professional associations and civil society organizations. Dr Nagral stressed the need for the healthcare sector to leverage public communication channels, such as media and political forums, to ensure that SAO care becomes a priority within electoral agendas. He also suggested the use of legal instruments, including public interest litigations and regulatory reforms, to draw attention to the SAO care infrastructure. In contrast, he noted that advocacy aimed at the general public must focus on building trust and raising awareness about SAO care services.

Dr Ghosh highlighted that the erosion of trust in healthcare systems remains a significant barrier to care-seeking behaviour, particularly in underserved communities [35, 36]. He stressed the importance of culturally appropriate communication strategies that resonate with local populations. Dr Preethi Kumar pointed to the Kerala model, where community-driven palliative care initiatives successfully empowered people to take ownership of their health [37]. She emphasized that similar bottom-up approaches, driven by the active participation of communities, could enhance SAO care awareness. Effective communication should focus on the social and emotional aspects of SAO care, ensuring that individuals understand the importance of timely interventions and feel empowered to seek necessary treatment.

Community health workers such as ASHAs play a crucial role in bridging the gap between healthcare systems and local communities [38]. Dr Keyur Buch highlighted the success of the SATHI project in Ahmedabad, where community health workers were trained to improve treatment-seeking behaviours in urban slums [39, 40]. He noted that these frontline workers can build trust within communities by addressing misconceptions about SAO care, early identification of surgical needs, and facilitating referrals to appropriate healthcare facilities. Their role extends beyond awareness, as they also contribute to health promotion by educating families about preventive measures and post-operative care, thus enhancing long-term health outcomes.

The discussion concluded with a consensus on the importance of tailored advocacy strategies to bridge gaps in SAO care delivery. Dr Kumar emphasized that the institutionalization of advocacy efforts through platforms such as the DIPICA Observatory could serve as a centralized resource for monitoring and surveillance, offering valuable insights to both policymakers and communities.

### SAO care policy reforms and frameworks

Dr Rajesh Mehta and Dr Preethi Kumar shed light on the critical need for policy-driven frameworks to address gaps in SAO care delivery in resource-constrained settings. Dr Mehta outlined the inequities prevalent in SAO care, particularly the challenges faced by rural populations in accessing essential and emergency procedures. He noted rural areas remain severely underserved despite advancements in healthcare infrastructure in urban centres, perpetuating the “law of inverse care,” where those in greatest need receive the least attention. Dr Mehta emphasized that SAO care must be included within the broader framework of UHC to achieve equity and financial protection. He proposed notable features of a sustainable and inclusive SAO care system, including the integration of SAO indicators into national health information systems to monitor performance and address disparities, tailoring state-level policies to address local health challenges, empowering community interest holders, and incorporating patient voices into policymaking.

Dr Mehta further identified key pillars for policy action, including workforce strengthening, improving essential infrastructure and supplies, quality assurance, and the strategic use of data for decision-making. He proposed leveraging technology and task-sharing models to address workforce shortages, particularly in underserved areas. However, he cautioned against diluting professional standards and emphasized the need for regulatory mechanisms to maintain safety and quality. Citing successful public health models, he argued for a phased approach to scaling up SAO care, beginning with foundational investments in primary and secondary care systems. He also underscored the role of data in framing policies, calling for improved collection and analysis of metrics such as SAO access, workforce distribution, and financial risk protection.

Building on this, Dr Kumar highlighted operational gaps and structural inefficiencies that hinder the implementation of SAO care in India. She pointed to the disproportionate burden of mortality due to road traffic accidents and unregulated highway expansion, which has exacerbated the need for timely trauma care. She also pointed out that the current medical curriculum does not adequately prepare graduates for surgical emergencies, particularly in rural settings. She proposed the development of trauma networks and referral systems to ensure timely surgical interventions and the incorporation of comprehensive surgical training at the undergraduate level to ensure that graduates are equipped to handle basic procedures and emergencies. Dr Kumar also emphasized the need for policies that empower community health officers with supportive policy and requisite skills to serve as first responders in rural areas, bridging the gap between the community and formal healthcare systems. Dr Kumar called for prioritizing investments in infrastructure and workforce training as essential components of strengthening SAO care. She noted that rural hospitals often lack basic facilities such as running water, electricity, essential surgical equipment, and oxygen, underscoring the need for targeted resource allocation.

Dr Mehta and Dr Preethi emphasized the role of community involvement in driving policy reforms, arguing that sustainable solutions must be co-designed with local interest holders. They highlighted the importance of leveraging technology, such as telemedicine, mobile health applications, and AI-driven solutions, to expand access to SAO care in underserved regions.

Dr Sonali Vaid emphasized the importance of collaboration and data-driven strategies to improve SAO care. She stressed the need for accessible, safe, and timely surgical services in underserved regions and underscored the role of novel approaches and technology in bridging gaps in SAO care delivery. Emphasizing her experience at the WHO Regional Office for Western Pacific (WPRO), which recently developed an action framework for safe and affordable surgery, she called for regional collaboration and knowledge sharing to address systemic challenges in low- and middle-income countries [41, 42]. Dr Vaid also pointed out that aligning national policies with global evidence-based standards would enhance equity and quality in care. She emphasised aligning the local advocacy efforts with ongoing global initiatives like WHO’s resolution WHA76.2, "Integrated emergency, critical and operative care for universal health coverage and protection from health emergencies” [43]. The attendees agreed that advocating for greater investment in health systems and community-driven initiatives to ensure sustainable progress in SAO care.

## Conclusion and future directions

The DIPICA Observatory Meeting marks a significant step toward advancing universal SAO care in India by fostering collaboration among care providers, public health experts, and policymakers. The discussions underscored the critical need for data-driven policymaking, emphasising the role of implementation research in overcoming workforce gaps, improving service delivery, and shaping effective interventions. By facilitating ongoing dialogue and promoting collaborative action, DIPICA aims to strengthen advocacy initiatives and drive sustainable change in SAO care access and quality.

**Fig. 3:**
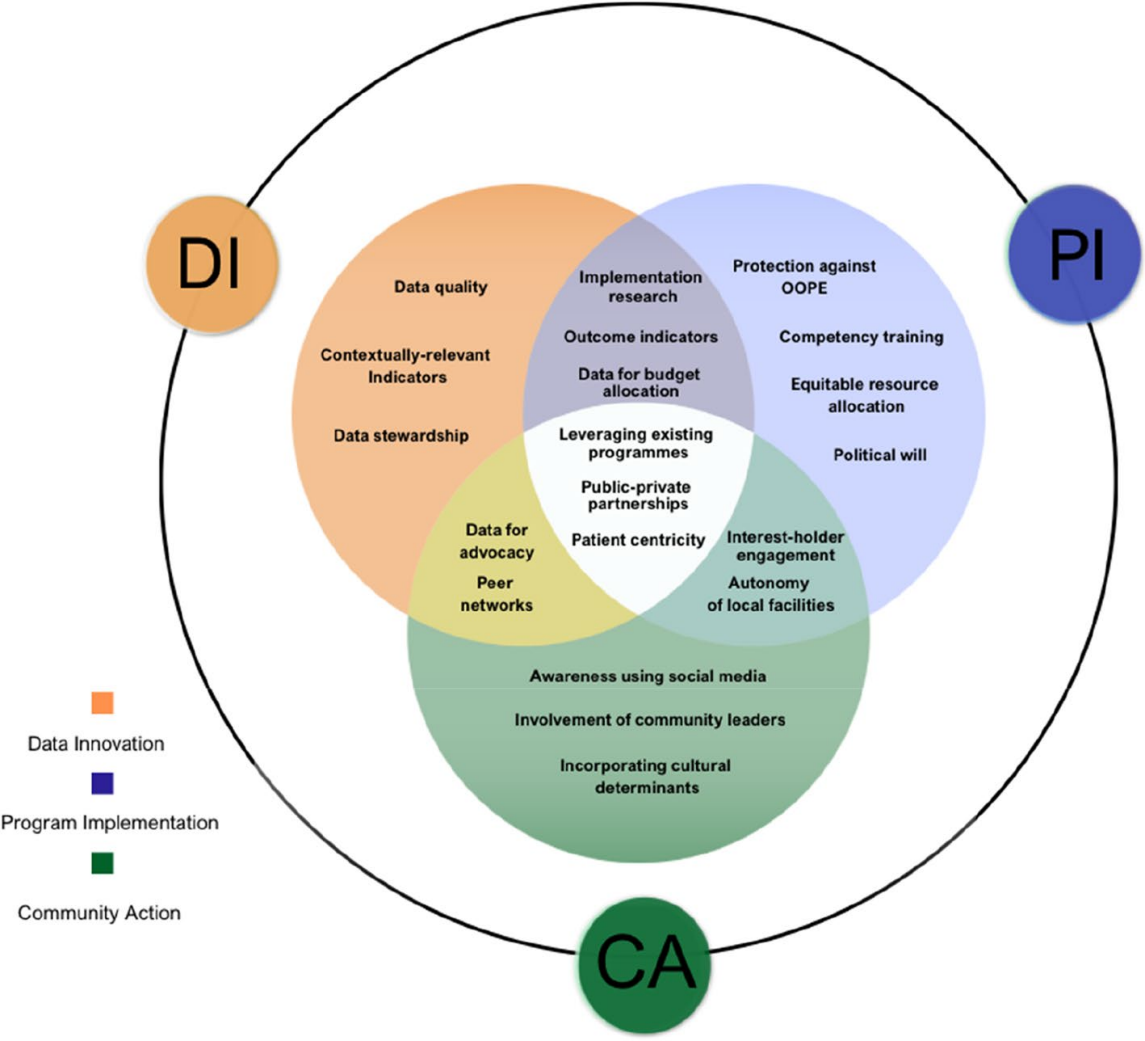
Venn Diagram showing prominent themes discussed at the DIPICA Meeting 2024

Figure 3 summarises the main actionable themes that emerged from the two-day DIPICA meeting across the Data Innovation (DI), Program Implementation (PI), and Community Action (CA) domains. Within DI, key themes included strengthening data quality, building contextual indicators, and ensuring privacy and security. PI themes included protection against out-of-pocket expenditure, competency training for existing healthcare providers, effective resource allocation, political will, and improving insurance coverage. CA focused on awareness through social media, involvement of community leaders, and addressing cultural determinants that influence access to and utilization of SAO care. Several cross-cutting priorities emerged across these domains. Between DI and PI, common themes included prioritizing implementation research, outcome indicators, and the use of data for appropriate budget allocations. Overlap of DI and CA focused on the use of data for advocacy, peer networks to support evidence use, and open access to information to ensure that those in low-resource settings are not punished. PI and CA shared emphasis on interest-holder engagement and autonomy for those running local healthcare facilities. Focusing on patient centricity in all research and practice, leveraging existing programmes, and judicious reliance on public–private partnerships were noted to be central to all three domains, highlighting the need for integrated efforts across data systems, programme delivery, and community engagement to strengthen SAO care in India.

SAO care financing in India demands comprehensive and innovative solutions. Currently, both patients and providers face significant financial barriers, which hinder access to essential surgical services. While some initiatives to improve financing exist in silos, there is an urgent need for a structured and cohesive SAO financing policy to achieve equitable and sustainable care. Integrating innovative models like low-cost innovative technologies in the health system infrastructure, strengthening existing frameworks of financial incentives under MoHFW, and fostering collaboration among government bodies like ICMR, NHSRC, NITI Aayog, the private sector, and international organisations will be crucial in addressing financial challenges.

The heterogeneity of healthcare providers and infrastructure exists across India, leading to disparities in access to and quality of SAO care. Addressing these disparities requires the development of context-specific policy frameworks that align with global benchmarks while reflecting India's ground reality. This can be achieved by upskilling the existing workforce, promoting task sharing, low-cost innovative technologies, and offering sustainable incentives to healthcare professionals and health centres in rural and remote areas. India can build a more resilient and efficient SAO care system by scaling successful local and global practices.

Effective advocacy for SAO care requires a multi-interest-holder approach, integrating community engagement, professional collaboration, and policymaker involvement. Moving forward, DIPICA aims to focus on gathering localized data through consultations and key informant interviews to better understand regional challenges and interest-holder perspectives. Developing a comprehensive data dashboard will support real-time monitoring and decision-making. Grassroots-level studies involving providers will help understand their needs and the facilitators and barriers to care. Securing funding to pilot and scale localized programs will be essential to translating research findings into actionable solutions. The DIPICA observatory also aims to serve as a platform for continuous dialogue and knowledge exchange, hosting annual meetings to review progress, share insights, and refine strategies. By fostering multi-sectoral collaboration and mobilizing financial resources, DIPICA is well-positioned to serve as a comprehensive roadmap to lead meaningful progress in achieving equitable, accessible, and high-quality SAO care across India.

## Supplementary Information

Supplementary Material 1.

## Data Availability

Not applicable.

## Abbreviations

DIPICA: Data Innovation, Program Implementation, and Community Action
NITI Aayog: National Institution for Transforming India Aayog
SPI: Surgical Preparedness Index
SAO: Surgical, Anaesthesia, and Obstetric
MBBS: Bachelor of Medicine and Bachelor of Surgery
PM-JAY: Pradhan Mantri Jan Arogya Yojana
HMIS: Health Information Management System
NFHS: National Family Health Survey
LCoGS: Lancet Commission on Global Surgery
AIIMS: All India Institute of Medical Sciences
GOI: Government of India
MoHFW: Ministry of Health and Family Welfare
ICMR: Indian Council of Medical Research
NHSRC: National Health System Resource Centre
WHO: World Health Organization
UHC: Universal Health Coverage
CEmONC: Comprehensive Emergency Obstetric and Neonatal Care
LSAS: Life Saving Anaesthesia Skills
