# Supplementary material for: Data Innovation, Program Implementation, and Community Action (DIPICA) Observatory for Surgical, Anesthesia, and Obstetric (SAO) Care in India
Source: BMC Proc. 2025 Nov 3;19(Suppl 31):35. doi: 10.1186/s12919-025-00351-3 (PMC12581580; doi:10.1186/s12919-025-00351-3)
Supplement: Supplementary file 1 — Supplementary Material 1. [file 12919_2025_351_MOESM1_ESM.docx]

**Additional file 1**

**Supplementary Table 1: Author list with details and contributions as per CRediT.**

| **Sr. No.** | **Name** | **Email address** | **Affiliations** | **Author Contributions** | **COI** |
| --- | --- | --- | --- | --- | --- |
| 1. | Shirish Rao | shirishrao.1608@gmail.com | Association for Socially Applicable Research (ASAR), Pune, Maharashtra, India - 411007; Seth G.S. Medical College and K.E.M. Hospital, Mumbai, Maharashtra, India - 400012; Global Alliance for Surgery, Obstetric, Trauma, and Anaesthesia Care, Chicago, Illinois USA - 60611 | Conceptualization, Methodology, Formal Analysis, Data Curation, Visualization, Project administration, Writing - Original Draft, Writing - Review & Editing | Nil |
| 2. | Uma Gupta | umagupta725@gmail.com | Association for Socially Applicable Research (ASAR), Pune, Maharashtra, India - 411007 | Conceptualization, Methodology, Formal Analysis, Data Curation, Visualization, Project administration, Writing - Original Draft, Writing - Review & Editing | Nil |
| 3. | Siddhesh Zadey | sidzadey@asarforindia.org | Association for Socially Applicable Research (ASAR), Pune, Maharashtra, India - 411007; Department of Surgery, Duke University of School of Medicine, Durham NC US - 27707; Dr. D.Y. Patil Medical College, Hospital, and Research Centre, Pune, Maharashtra, India - 411018; Global Alliance for Surgery, Obstetric, Trauma and Anaesthesia Care, Chicago, Illinois USA - 60611 | Conceptualization, Methodology, Visualization, Resources, Project administration, Writing - Original Draft, Writing - Review & Editing, Supervision, | Siddhesh Zadey is the co-founding director of the Association for Socially Applicable Research (ASAR). He also represent ASAR at the G4 Alliance Permanent Council. He serves as the Chair of the SOTA Care in Asia Working Group, The G4 Alliance, Fellow of the Lancet Citizens’ Commission on Reimagining India’s Health System, and the Drafting Committee Member for Maharashtra State Mental Health Policy. |
| 4. | Lovenish Bains | lovenishbains@gmail.com | Department of Surgery, Maulana Azad Medical College & Lok Nayak Hospital, New Delhi- 110002, India | Conceptualization, Methodology, Resources, Supervision, Project Administration, Writing – review & editing | Nil |
| 5. | Dhruva Ghosh | dhruvghosh73@gmail.com | India Hub, NIHR Health Research Unit On Global Surgery, Christian Medical College, Ludhiana, Punjab, India- 141008 | Conceptualization, Methodology, Resources, Supervision, Project Administration, Writing – review & editing | Nil |
| 6. | Joao Vissoci | jnv4@duke.edu | GEMINI Research Center, Duke University School of Medicine, Durham, NC, USA | Conceptualization, Methodology, Resources, Supervision, Project Administration, Writing – review & editing, Funding aquisation | Nil |
| 7. | Aaradhana Vaghela | aaradhanavaghela1@gmail.com | Association for Socially Applicable Research (ASAR), Pune, Maharashtra, India - 411007 | Writing – review & editing | Nil |
| 8. | Aiman Perween Afsar | afsaraiman@gmail.com | Association for Socially Applicable Research (ASAR), Pune, Maharashtra, India - 411007 | Writing – review & editing | Nil |
| 9. | Anchal Dhiman | anchal.dhiman@nhsrcindia.org | Consultant, Public Health Administration Division, National Health Systems Resource Centre (NHSRC) | Writing – review & editing | Consultant, National Health Systems Resource Centre (NHSRC) |
| 10. | Anoushka Arora | anoushkaarora.arora@gmail.com | Association for Socially Applicable Research (ASAR), Pune, Maharashtra, India - 411007 | Writing – review & editing | Nil |
| 11. | Anurag Mishra | profanuragmamc@gmail.com | Department of Surgery, Maulana Azad Medical College | Writing – review & editing | Nil |
| 12. | Chaitanya Reddy | chaitureddy2810@gmail.com | Association for Socially Applicable Research (ASAR), Pune, Maharashtra, India - 411007 | Writing - Original Draft, Writing – review & editing | Nil |
| 13. | Chinmayee Swain | dr.chinmayeeswain@gmail.com | Consultant, Quality & Patient Safety, National Health Systems Resource Centre (NHSRC) | Writing – review & editing | Consultant, National Health Systems Resource Centre (NHSRC) |
| 14. | Gnanaraj Jesudian | jgnanaraj@gmail.com | SSIPMT; Association of Rural Surgeons of India (ARSI) | Writing – review & editing | Nil |
| 15. | Harsh Thakkar | harshnj27@gmail.com | Association for Socially Applicable Research (ASAR), Pune, Maharashtra, India - 411007 | Writing – review & editing | Nil |
| 16. | Janet Martin | jmarti83@uwo.ca | Anesthesia & Perioperative Medicine, Western University | Writing – review & editing | Janet Martin is a consultant to WHO and WFSA; Advisory roles with NIHR Global Surgery Unit, Utstein Group, WFSA, Laerdal Foundation. |
| 17. | Kalpana Pawalia | kalpana.pawalia@nhsrcindia.org | Consultant, National Health Systems Resource Centre (NHSRC) | Writing – review & editing | Consultant, National Health Systems Resource Centre (NHSRC) |
| 18. | Kapil Dev Soni | kdsoni111@gmail.com | Professor, Critical and Intensive Care, JPN Apex Trauma Centre, AIIMS, New Delhi | Writing – review & editing | Nil |
| 19. | Keyur Buch | k3buch@gmail.com | Hon. Adj. Associate Professor, Bond University Medical School; Consultant Orthopedic & Trauma Surgeon, Epic Hospital | Writing – review & editing | Nil |
| 20. | Lalit Gupta | lalit.doc@gmail.com | Professor, Anaesthesia, Maulana Azad Medical College | Writing – review & editing | Nil |
| 21. | Madhurima Vuddemarry | madhurimav283@gmail.com | Association for Socially Applicable Research (ASAR), Pune, Maharashtra, India - 411007 | Writing – review & editing | Nil |
| 22. | Maithili Kukade | milikukade@gmail.com | Association for Socially Applicable Research (ASAR), Pune, Maharashtra, India - 411007 | Writing – review & editing | Nil |
| 23. | Naveen Sharma | drnsemail@gmail.com | Department of General Surgery, AIIMS Jodhpur | Writing – review & editing | Nil |
| 24. | Nishikant Singh | nishiiips@gmail.com | Health Systems Transformation Platform | Writing – review & editing | Nil |
| 25. | Ojaswi Phal Desai | ojaswiphaldessai@gmail.com | Association for Socially Applicable Research (ASAR), Pune, Maharashtra, India - 411007 | Visualization, Writing – review & editing | Nil |
| 26. | Padmavathy Krishna Kumar | padbhukrish@gmail.com | Association for Socially Applicable Research (ASAR), Pune, Maharashtra, India - 411007;Department of Epidemiology, Mailman School of Public Health, NYC, USA | Writing – review & editing | Nil |
| 27. | Pratheeba John | pratheebaj@gmail.com | Health Systems Transformation Platform (HSTP), New Delhi | Writing – review & editing | Nil |
| 28. | Preeti Kumar | preeti.kumar@phfi.org | Public Health Foundation of India | Writing – review & editing | Nil |
| 29. | Priyansh Nathani | priyanshnathani@gmail.com | WHO Collaborating Centre for Emergency, Critical and Operative Care, The George Institute for Global Health, New Delhi | Writing – review & editing | Nil |
| 30. | Rahul M. Jindal | jindalr@msn.com | Professor, Department of Surgery & Global Health, Uniformed Services University | Writing - Original Draft, Writing – review & editing | Nil |
| 31. | Rajesh Mehta | drrajeshmehta@gmail.com | Adjunct Professor, Public Health Foundation of India | Writing – review & editing | Nil |
| 32. | Rajna Mishra | rajna.mishra@phfi.org | Assistant Director – Health Systems; Adjunct Professor, Public Health Foundation of India | Writing – review & editing | Nil |
| 33. | Rakesh Garg | drrgarg@hotmail.com | Professor, All India Institute of Medical Sciences, New Delhi | Writing – review & editing | Nil |
| 34. | Ritika Shetty | ritika.a2799@gmail.com | Association for Socially Applicable Research (ASAR), Pune, Maharashtra, India - 411007 | Writing – review & editing | Nil |
| 35. | Rituparna Sengupta | rsrsengupta230@gmail.com | Independent Researcher | Writing – review & editing | Nil |
| 36. | Rodney Preetham Vaz | rodneyvaz1993@gmail.com / rodneypreethamv@gmail.com | Assistant Professor of Community Medicine, SGT University; Associate Fellow, Foundation for People-Centric Health Systems | Writing – review & editing | Nil |
| 37. | Samruddha Kulkarni | worksamruddha@gmail.com | Association for Socially Applicable Research (ASAR); Seth G.S. Medical College & KEM Hospital | Writing - Original Draft, Writing – review & editing | Nil |
| 38. | Sanjay Nagral | sanjaynagral@gmail.com | Jaslok Hospital and Research Centre, Mumbai | Writing – review & editing | Nil |
| 39. | Shikha Sharma | shikha.sharma@nhsrcindiaextn.org | Consultant, National Health Systems Resource Centre (NHSRC) | Writing – review & editing | Consultant, National Health Systems Resource Centre (NHSRC) |
| 40. | Shreyas Patil | patil19.shreyas@gmail.com | Association for Socially Applicable Research (ASAR); Senior Resident, Community Medicine & Family Medicine, AIIMS Bibinagar | Writing – review & editing | Nil |
| 41. | Sudheer Kumar Shukla | sudheer.iips@gmail.com | Health Systems Transformation Platform (HSTP), New Delhi | Writing – review & editing | Nil |
| 42. | Suraj Bhor | suraj.bhor@tuebingen.mpg.de | Max Planck Institute for Intelligent Systems, Tübingen, Germany | Writing – review & editing | Nil |
| 43. | Tej Prakash Sinha | drsinha123@gmail.com | Additional Professor, Department of Emergency Medicine, JPNATC, AIIMS, New Delhi | Writing – review & editing | Nil |
| 44. | Tushar S. Mishra | doctushar@gmail.com | Department of Surgery, AIIMS Bhubaneswar | Writing – review & editing | Nil |
